# Low renal replacement therapy incidence among slowly progressing elderly chronic kidney disease patients referred to nephrology care: an observational study

**DOI:** 10.1186/s12882-017-0473-1

**Published:** 2017-02-10

**Authors:** Ulrika Hahn Lundström, Alessandro Gasparini, Rino Bellocco, Abdul Rashid Qureshi, Juan-Jesus Carrero, Marie Evans

**Affiliations:** 1 Division of Renal Medicine, Department CLINTEC, Karolinska Institutet and Karolinska University Hospital, Stockholm, Sweden; 2 Department of Statistics and Quantitative Methods, University Milano-Bicocca, Milan, Italy; 3 Department of Medical Epidemiology and Biostatistics, Karolinska Institutet, Stockholm, Sweden; 4 Center for Molecular Medicine, Karolinska Institutet, Stockholm, Sweden; 5 Renal Department M99, Karolinska University Hospital Huddinge, Stockholm, SE-14186 Sweden

**Keywords:** Renal replacement therapy, Chronic Kidney Disease, Estimated glomerular filtration rate, Mortality, End-stage kidney disease, Progression rate, Epidemiology

## Abstract

**Background:**

Elderly patients with advanced chronic kidney disease (CKD) have a high risk of death before reaching end-stage kidney disease. In order to allocate resources, such as advanced care nephrology where it is most needed, it is essential to know which patients have the highest absolute risk of advancing to renal replacement therapy (RRT).

**Methods:**

We included all nephrology-referred CKD stage 3b-5 patients in Sweden 2005–2011 included in the Swedish renal registry (SRR-CKD) who had at least two serum creatinine measurements one year apart (+/− 6 months). We followed these patients to either initiation of RRT, death, or September 30, 2013. Decline in estimated glomerular filtration rate (eGFR) (%) was estimated during the one-year baseline period. The patients in the highest tertile of progression (>18.7% decline in eGFR) during the initial year of follow-up were classified as “fast progressors”. We estimated the cumulative incidence of RRT and death before RRT by age, eGFR and progression status using competing risk models.

**Results:**

There were 2119 RRT initiations (24.2%) and 2060 deaths (23.5%) before RRT started. The median progression rate estimated during the initial year was −8.8% (Interquartile range [IQR] - 24.5–6.5%). A fast initial progression rate was associated with a higher risk of RRT initiation (Sub Hazard Ratio [SHR] 2.24 (95% confidence interval [CI] 2.00–2.51) and also a higher risk of death before RRT initiation (SHR 1.27 (95% CI 1.13–1.43). The five year probability of RRT was highest in younger patients (<65 years) with fast initial progression rate (51% in CKD stage 4 and 76% in stage 5), low overall in patients >75 years with a slow progression rate (7, 13, and 25% for CKD stages 3b, 4 and 5 respectively), and slightly higher in elderly patients with a fast initial progression rate (28% in CKD stage 4 and 47% in CKD stage 5) or with diabetic kidney disease.

**Conclusions:**

The 5-year probability of RRT was low among referred slowly progressing CKD patients >75 years of age because of the competing risk of death.

**Electronic supplementary material:**

The online version of this article (doi:10.1186/s12882-017-0473-1) contains supplementary material, which is available to authorized users.

## Background

Chronic kidney disease (CKD) is a growing health problem; the population prevalence has increased to an estimated 13% in the United States [1]. Although CKD affects all ages there is a strong positive association with age: in individuals over 70 years, prevalence is almost 50% [2]. With an aging population, it may be difficult to identify which patients would benefit most from referral to and follow-up by a nephrologist [3]. Many diagnosed with stable CKD stage 3 can be followed up by primary health care after the initial nephrology evaluation. However, patients with advanced kidney disease and a high risk for renal replacement therapy (RRT) may benefit from nephrology care to facilitate the necessary preparations for RRT such as timely fistula surgery, if consistent with their preferences.

One risk factor for both RRT and death is a rapid decline in renal function. It was recently found that patients with renal function loss >30% over an initial period of two years had a higher risk of end-stage renal disease (ESRD) regardless of age and estimated glomerular filtration rate (eGFR) [4]. However, this risk may differ in the “real-life” setting of unselected patients referred to nephrology care as opposed to patients included in various studies and general population cohorts. Many elderly patients can after a drop in eGFR, have stable eGFR levels for several years without the need for dialysis initiation [5, 6]. In this study we aimed to investigate the absolute risk of RRT associated with a slow versus rapid disease progression in a nationally representative sample of nephrology-referred patients taking into account the competing risk of death.

## Methods

### Study population

**Fig. 1:**
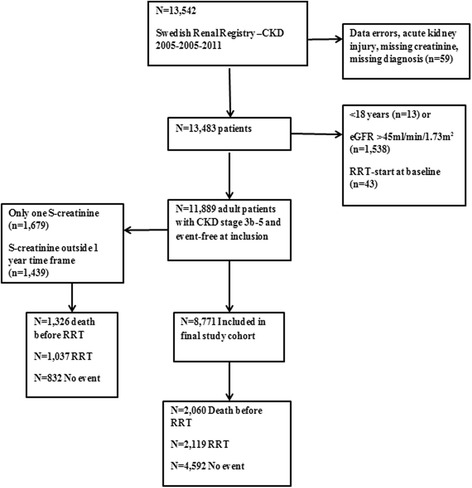
Patient’s inclusion flow chart. RRT (renal replacement therapy), eGFR (estimated glomerular filtration rate), N (number), CKD (chronic kidney disease)

The Swedish Renal Registry (SRR) is one of the national health data registries used to evaluate, control and compare health care quality across the country [7, 8]. Swedish Renal Registry - Chronic Kidney Disease (SRR-CKD) is the part of SRR that includes patients referred to nephrology clinics throughout Sweden. Clinics are urged to register patients when the renal function deteriorates ≤ 30 mL/min/1.73 m^2^ with the option to register earlier if the local center so wishes. We included all patients in the SRR-CKD, with eGFR <45 ml/min/1.73 m^2^ and a baseline visit between January 1, 2005 and December 31 2011. Patients were followed until death, RRT-initiation or September 30, 2013, whatever came first. At inclusion, patients received written information about SRR and were permitted to refuse participation in the registry. According to Swedish law, written consent is not required because quality control is an inherent element of hospital health care. The Regional Ethics committee in Stockholm approved the study protocol. The patient’s flow chart is presented in Fig. 1. The study adhered to the STROBE criteria for observational cohort studies (Additional file 1).

### Renal function decline

The vast majority of the creatinine measurements (98%) were performed by enzymatic or corrected Jaffe method both of which are traceable to isotope dilution mass spectrometry standards [9]. GFR was estimated from serum creatinine by the CKD-EPI equation [10] and stratified per CKD stages. Progression rate was measured as the difference (in %) between the first and last eGFR during a one-year baseline period. For the second measurement, we selected the serum creatinine closest to one-year allowing measurements to vary within a six months range (thus the baseline year ranged from 6 to 18 months). The values were then rescaled to express the yearly % change. Patients without a second serum creatinine within this period were excluded. Patients with a progression rate in the highest tertile were considered “fast progressors” while all others were considered “slow progressors”.

### Clinical measures

The baseline measurements used for this analysis were all obtained from the first visit of the follow-up, which occurred after the initial one-year period when eGFR decline was estimated (Additional file 2: Figure S1). The treating nephrologist assigned the primary renal diagnosis; additional comorbidity was obtained through linkage with the National Patient Registry collecting information from all hospitalizations in Sweden. Comorbidity was further classified into a Charlson comorbidity index score [11]. Since all the patients had CKD, the minimum score was 2. Therefore, we centered the score on the value 2, giving the score the meaning of additional comorbidities to CKD. Blood pressure and body mass index (BMI) were registered at the first out-patient visit, together with the prescribed medications and treatment with a protein restricted diet (≤0.6 g/kg/day). All laboratory measurements were collected as a part of the regular clinical follow-up at different clinical chemistry laboratories throughout Sweden. Although no formal calibration was performed, the laboratories were all monitored regularly by Equalis Sweden to assess the quality standards of the different analytical methods [9, 12].

### Statistical analysis

Age was categorized as < 65, 65–75, and ≥ 75 years of age, eGFR was categorized in CKD stages, BMI was categorized according to WHO’s definition (<18.5, 18.5–25, 25–30, >30 kg/m^2^), calcium and phosphate were included in categories of 0.1 units increase, while pulse pressure and mean arterial pressure were used in categories of 10 mmHg increase. Remaining continuous covariates were included assuming a linear effect on the outcome. There was a varying proportion of missingness in our data. Most data had missing values <10% whereas albumin-creatinine ratio (ACR) had up to 65% missing and was only considered in sensitivity analyses. We performed multiple imputation for variables with up to 30% of missing values using multiple imputation with chained equation [13]. We imputed numeric variables using predictive mean-matching and binary response variables using Bayesian logistic regression. The algorithm iterated 30 times, and convergence was satisfactory. Finally, 55 datasets were imputed and goodness of fit of the whole procedure was evaluated and found to be good.

Being in a competing risks framework, the occurrence of the event “death before RRT” impedes the occurrence of the event “RRT initiation” and vice versa. Therefore, we used ad hoc methods to analyze both outcomes. First, we used Cox semiparametric models with Efron’s method for ties to analyze the cause-specific hazards treating subjects who experienced the competing event as censored. Subsequently, we analyzed the sub-hazards using Fine & Gray models [14] which were adjusted to account for both left truncation and right censoring [15]. We analyzed cause-specific hazards and sub-hazards jointly with regard to both outcomes in order to have a complete understanding of the event dynamics, and of the association of the covariates with cause-specific hazards and cumulative incidence functions [16]. The included variables were those recorded at the first follow-up visit after the initial one-year period, including eGFR at inclusion and CKD-stage. Interactions between age class, CKD stage and disease progression were evaluated; significant interactions were retained in the models used to estimate cumulative incidence functions of event, discarded when presenting relative risk estimates in order to ease interpretation. Goodness of fit of the models was also evaluated, with particular attention paid to the underlying proportionality assumption. No violations of the assumption were found in our analyses.

Finally, cause-specific probabilities of event at five years were estimated from the cause-specific Cox models for every possible combination of age class, CKD stage and disease progression, and both outcomes, adjusting for the median value (or most frequent, if categorical) of the remaining covariates included in the models (male gender, presence of hypertensive/ renovascular kidney disease, BMI of 25–30 kg/m2, non-university clinic, no erythropoietin stimulating agent use, no low protein diet, no statin or iron use, use of diuretics and vitamin D, plus median value of remaining laboratory data). These estimates were produced for individuals with diabetic kidney disease as well, everything else being equal. Analogously, cumulative incidence function curves were produced using estimated coefficients from the Fine & Gray models for every possible combination of age class, CKD stage, disease progression and outcome, adjusted for the remaining covariates using their median or most frequent value. The analyses were performed using Stata 14.2 (StataCorp LP, http://www.stata.com) and R 3.3.2 (http://www.r-project.org).

## Results

### Study cohort characteristics

There were 13,542 patients with an outpatient visit during the defined time-period. We excluded 1,653 patients with age < 18 years, data errors, acute kidney injury or eGFR ≥ 45 ml/min/1.73 m^2^. Of the remaining 11,889 patients, another 3,118 patients were excluded because of missing progression rate (follow-up less than one year or only one serum creatinine measurement within the pre-specified time frame) (Fig. 1). The remaining cohort consisted of 8,771 patients with a mean age of 72 years and of whom 64% were men. Most of the patients were diagnosed with hypertensive kidney disease (26%), followed by diabetes nephropathy (20%), glomerulonephritis (10%), other specified causes (21%) and unknown cause of uremia (23%). The majority of the patients were in CKD stage 4 and the median eGFR was 20.2 ml/min/1.73 m^2^ after the initial one-year baseline period.

### Progression rate

The median progression rate estimated during the initial year was −8.8% (Interquartile range [IQR] -24.5 − 6.5%) or an absolute median decline by −1.71 ml/min/1.73 m^2^ (IQR −4.81 – 1.33). Even though most of the patients experienced deteriorating renal function, a substantial proportion (35.1%) had no change or an improvement in eGFR during the first year. Patients defined as fast progressors (highest tertile, > −18.7% decline/year) were younger, had lower eGFR at baseline, lower hemoglobin, higher ACR, and higher phosphate compared with patients who were defined as non-progressors (Table 1).

**Table 1:** Demographics by disease progression in a national, representative cohort of referred patients with chronic kidney disease

Demographics	Progressors *N* = 2,924 (33.3%)	Non-progressors *N* = 5,847 (66.6%)
Age, median	69.9 (59.3–78.7)	73.4 (64.2–80.6)
<65 years	1,091 (37.3%)	1,568 (26.8%)
65- < 75 years	767 (26.2%)	1,683 (28.8%)
≥75 years	1,066 (36.5%)	2,596 (44.4%)
Men	1,931 (66.0%)	3,670 (62.8%)
Primary renal disease		
Hypertension/renovascular	732 (25.0%)	1,538 (26.3%)
Diabetes nephropathy	721 (24.7%)	1,071 (18.3%)
Glomerulonephritis	315 (10.8%)	600 (10.3%)
Other specified disease	618 (21.1%)	1,199 (20.5%)
Unknown	538 (18.4%)	1,439 (24.6%)
Progression rate (% decline in ml/min/1.73 m^2^ per year)	−31.9 (−43.9––24.5)	0.60 (−8.8–14.6)
Progression rate (absolute change in ml/min/1.73 m^2^ per year)	−6.1 (−8.6––4.3)	0.13 (−1.8–3.0)
Chronic Kidney Disease Stage		
G3b	56 (1.9%)	1,434 (24.5%)
G4	1,117 (38.2%)	3,570 (61.1%)
G5	1,751 (59.9%)	843 (14.4%)
Comorbidity at baseline		
Diabetes	1,172 (40.1%)	2,118 (36.2%)
Cardiovascular disease	1,109 (37.9%)	2,205 (37.7%)
Dementia	24 (0.8%)	57 (1.0%)
Chronic pulmonary disease	217 (7.4%)	483 (8.3%)
Cancer	414 (14.2%)	1,011 (17.3%)
Charlson score above kidney disease median (IQR)	1.0 (0.00–2.0)	1.0 (0.0–2.0)
Current medication		
ESA use [*n* = 7221]	680 (23.3%)	1,062 (18.2%)
Antihypertensives number of, median (IQR)	3.0 (2.0–4.0)	3.0 (2.0–4.0)
Protein restricted diet use	436 (14.9%)	545 (9.3%)
Diuretics use	2,148 (73.5%)	3,974 (68.0%)
Statin use	1,311 (44.8%)	2,727 (46.6%)
Vitamin-D supplements use	1,770 (60.5%)	3,005 (51.4%)
Iron use		
intravenous	286 (9.8%)	425 (7.3%)
oral	524 (17.9%)	853 (14.6%)
Laboratory data		
P-Albumin (g/l): [*n* = 8177]	36.0 (33.0–39.0)	37.0 (35.0–40.00=)
S-Calcium (mmol/l): [*n* = 7578]	2.28 (2.17–2.37)	2.31 (2.23–2.39)
CRP (mmol/l): [*n* = 5723]	5.0 (2.8–10.0)	5.0 (2.5–10.0)
P-Phosphate (mmol/l): [*n* = 7997]	1.5 (1.3–1.8)	1.2 (1.1–1.4)
S-Creatinine (mmol/l):	355 (272–475)	217 (177–268)
B-Hemoglobin (g/l): [*n* = 8537]	117 (108–127)	125 (116–135)
S-PTH (ng/ml): [*n* = 5818]	21.0 (12.9–33.7))	14.2 (9.5–21.8)
U-Albumin/creatinine ratio (mg/mmol) [*n* = 3149]	40.1 (9.4–170.9)	12.0 (2.5–48.0)
eGFR (ml/min/1.73 m^2^):	13.3 (9.4–18.2)	23.6 (18.0–29.9)
Clinical information		
Mean arterial pressure (median mmHg, IQR) [*n* = 8159]	97.6 (90.0–106.6)	96.0 (88.3–103.3)
Pulse pressure (median mmHg, IQR) [*n* = 8159]	60.0 (50.0–75.0)	60.0 (49.0–70.0)
BMI (IQR) [*n* = 6432]		
<18.5 Kg/m^2^	27 (0.9%)	61 (1.0%)
18.5–25 Kg/m^2^	733 (25.1%)	1,344 (23.0%)
25–30 Kg/m^2^	781 (26.7%)	1,630 (27.9%)
>30 Kg/m^2^	640 (21.9%)	1,216 (20.8%)
Region		
Lower North	676 (23.1%)	1,319 (22.6%)
South	405 (13.9%)	777 (13.3%)
Southeast	356 (12.2%)	600 (10.3%)
Southwest	514 (17.6%)	1,166 (19.9%)
Upper North	151 (5.2%)	269 (4.6%)
Stockholm	822 (28.1%)	1,716 (29.4%)
University Hospital	1,192 (40.8%)	2,381 (40.7%)

All categorical values are expressed as numbers (n) and percentage (%). All continuous variables are expressed as median and interquartile range (IQR). Erythropoietin stimulating agents (ESA), estimated glomerular filtration rate (eGFR), Serum (S), Plasma (P), Blood (B), and Urine (U). To convert Calcium in mmol/l to mg/dL divide by 0.2495. To convert Phosphate mmol/l to mg/dL multiply with 3.0974. To convert Hemoglobin in g/l to g/dL divide by 10. To convert creatinine from micromole/l to mg/dL multiply by 0.0113

### Mortality

**Fig. 2:**
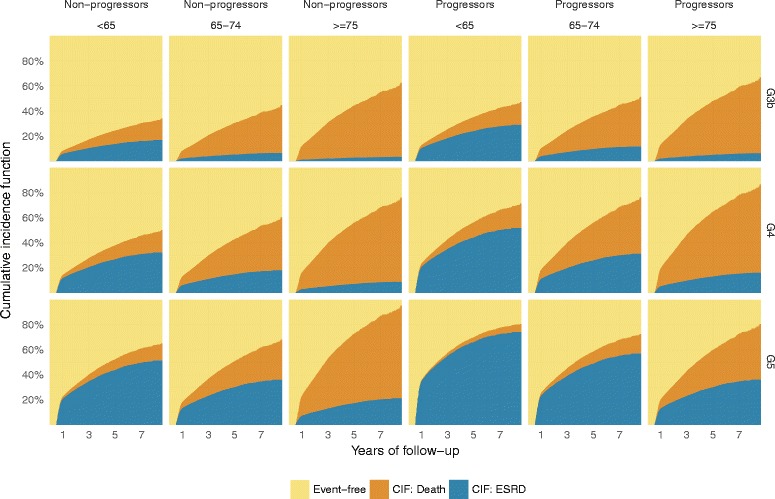
Cumulative incidence function for renal replacement therapy (blue) and death before renal replacement therapy (orange) by age, chronic kidney disease stage, and disease progression rate for patients under nephrology care. The cumulative incidence function was adjusted for the remaining covariates using their median value if numeric or most frequent value if categorical. On the Y-axis is cumulative incidence stratified by three CKD stages. On the X-axis is the time from inclusion in years, stratified by age category and progression status after the initial baseline period

Median follow-up time after the initial year was 2.8 years, (IQR 1.7 − 3.9 years). There were 2,060 deaths (23.5%) recorded before RRT was initiated during follow-up. Patients >75 years of age had almost six times higher risk of death before RRT was initiated compared with patients less than 65 years (Table 2). Variables associated with lower risk of death before RRT were female sex, BMI > 30 kg/m^2^ and having diabetic kidney disease or glomerulonephritis compared with hypertensive kidney disease. Patients classified as “fast progressors” had a higher pre-dialysis mortality (Sub-hazard ratio [SHR] 1.27; 95% CI 1.13–1.43). However, the absolute risk of death before RRT was not very different in fast progressors as compared with slow progressors except for patients > 75 years of age with CKD stage G5 (Fig. 2).

**Table 2:** Sub hazard ratios for initiation of renal replacement therapy and death before initiation of renal replacement therapy in a nationwide representative referred cohort of chronic kidney disease patients

Demographics	RRT initiation			Death before RRT initiation		
	SHR	95% CI	*P*-value	SHR	95% CI	*P*-value
Age (years)						
<65	1.00	ref		1.00	ref	
65–75	0.64	0.57–0.72	<0.01	2.48	2.07–2.97	<0.01
>75	0.449	0.40–0.51	<0.01	5.47	4.61–6.49	<0.01
Women	0.72	0.66–0.80	<0.01	0.77	0.70–0.85	<0.01
Primary renal disease						
Hypertensive kidney disease	1.00	ref		1.00	ref	
Diabetes nephropathy	1.21	1.03–1.41	0.020	0.57	0.45–0.72	<0.01
Glomerulonephritis	1.04	0.91–1.19	0.59	0.78	0.67–0.90	<0.01
Other specified disease	0.67	0.57–0.78	<0.01	1.06	0.94–1.19	0.36
Unknown	1.00	0.97–1.04	0.87	1.20	1.17–1.24	<0.01
Fast progressor (above 18.7% decline/year)	2.24	2.00–2.51	<0.01	1.27	1.13–1.43	<0.01
CKD stage^a^						
G3b	1.00	ref		1.00	ref	
G4	2.04	1.52–2.75	<0.01	0.94	0.80–1.09	0.40
G5	4.05	2.89–5.67	<0.01	0.94	0.75–1.19	0.61
Charlson Score above kidney disease (per 1 unit increase)	1.15	1.04–1.28	0.01	1.06	0.95–1.17	0.31
Body Mass Index (kg/m^2^)						
<18.5	1.08	0.72–1.62	0.72	1.39	0.98–1.96	0.06
18.5–25	1.00	ref		1.00	ref	
25–30	0.91	0.81–1.02	0.11	0.96	0.85–1.07	0.46
>30	0.90	0.80–1.02	0.11	0.86	0.76–0.98	0.02

Chronic Kidney Disease (CKD) Renal replacement therapy (RRT), Sub-hazard ratio (SHR) Confidence interval (CI). All variables, estimates are adjusted for all other variables, eGFR after the initial follow-up, current medication, laboratory data, hospital status, region and diet

^a^after the initial one-year baseline period when progression rate was estimated

### Initiation of renal replacement therapy

Almost one-fourth (24.2%) of all patients initiated RRT while 52.4% were event-free at the end of the follow-up period. Taking the competing risk of death into account the most important risk factors of RRT initiation was lower CKD stage, lower age, and a fast one-year progression rate (Table 2). Higher comorbidity score and diabetic kidney disease relative to hypertensive kidney disease was associated with higher RRT incidence. Also, women had a much lower incidence of RRT (SHR 0.72; 95% CI 0.66–0.80) compared with men in the extensively adjusted final model.

**Fig. 3:**
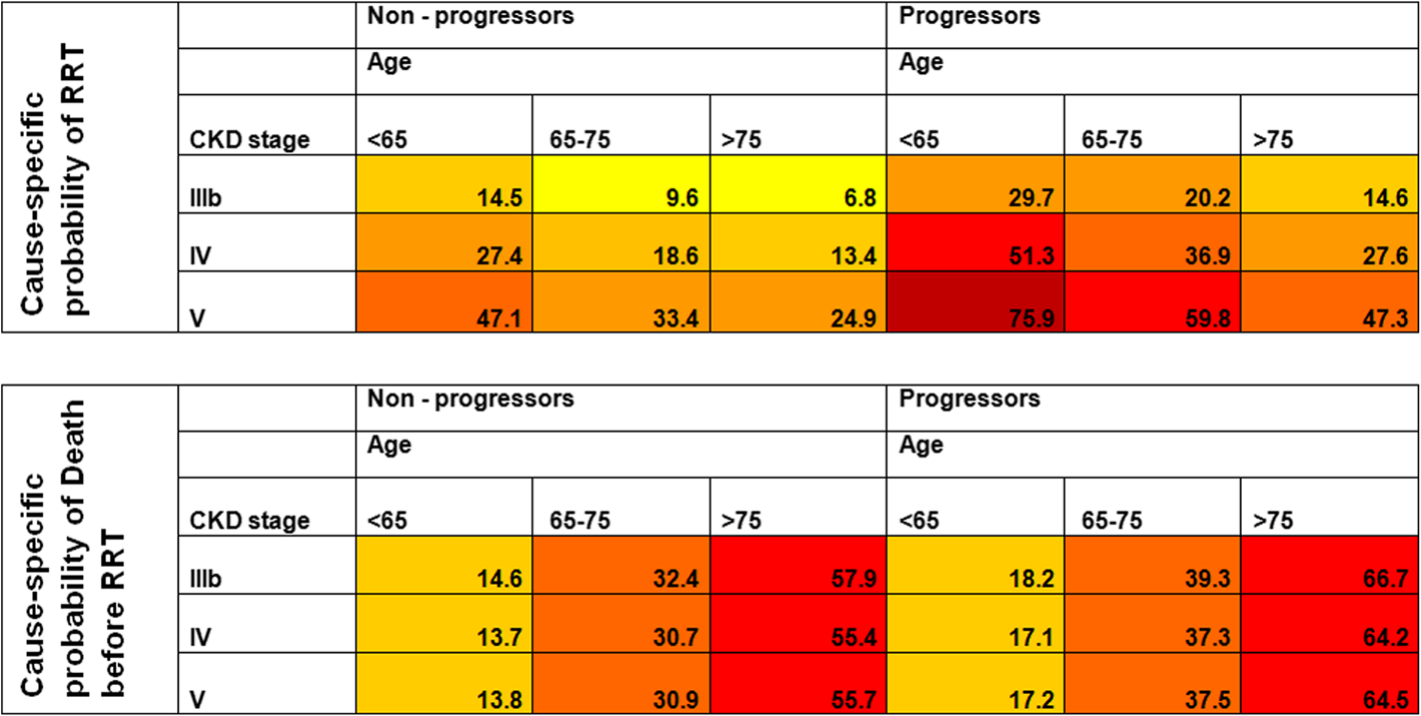
Five-year cause-specific probabilities of renal replacement therapy and death before renal replacement therapy for hypertensive kidney disease patients with a fast and slow progression rate stratified by age and chronic kidney disease stage. CKD (chronic kidney disease) categorized according to KDIGO classification system

**Fig. 4:**
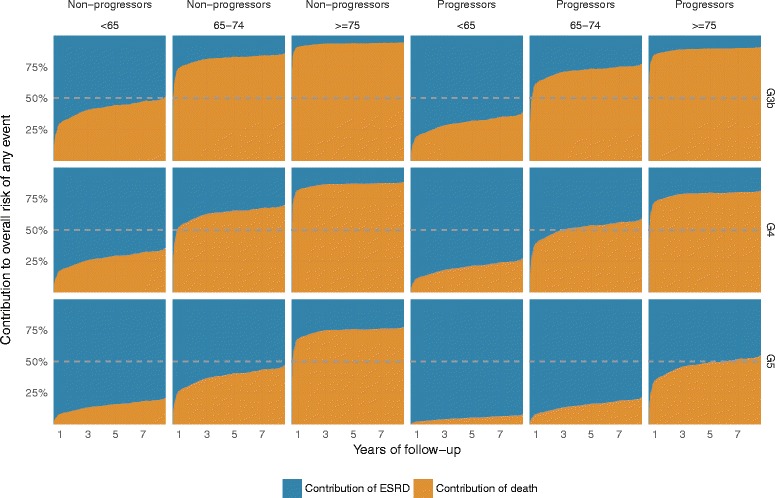
Contribution of each competing event (renal replacement therapy and death) to the overall risk of an event at any time during follow-up. The area represents the contribution of each competing event to the overall risk of event at any time. The dashed horizontal line at 50% is the equivalence of contributions line

The cumulative risk of RRT decreased by age and increased by CKD stage among both slow and fast progressors (Fig. 2). Patients characterized as fast progressors had a markedly higher cumulative risk of RRT in all age groups and CKD stages. The only patients in the slowly progressing group who had close to 50% cause-specific risk of RRT within five years were those younger than 65 years in stage 5 (Fig. 3), while among fast progressors even higher risk was observed among all patients <75 years (stage 5) and <65 years (stage 4–5). Slowly progressing patients above 75 years of age had a low risk of RRT regardless of CKD stage (25% for patients in CKD stage 5, 13% for patients in CKD stage 4), while elderly (>75 years) fast progressors in CKD stage 5 had a 47% risk of RRT within 5 years. Even though diabetic patients had a higher risk of RRT compared with patients with hypertensive kidney disease (SHR of 1.33, 95% C.I. of 1.13–1.56), this did not translate into a big difference in absolute risk of RRT (Additional file 2: Figure S2). Patients who were >75 years with a slow progression rate always had greater risk for death than start of RRT regardless of renal function, while patients <65 years of age had higher or equally high risk of RRT compared with death before RRT regardless of renal function and progression rate (Fig. 4).

Finally, we performed sensitivity analyses in patients with information on albuminuria: the overall coefficients from both cause-specific and competing risks models were consistent with the main analysis for both mortality and risk of RRT.

## Discussion

In this nationwide study of unselected, representative nephrology care patients in Sweden with eGFR <45 ml/min/1.73 m^2^ we found that clinical characteristics such as age, sex and CKD stage greatly influenced the risk of RRT and mortality before RRT initiation. Further, the preceding one-year progression rate was an important indicator of the future RRT risk. In general, elderly patients classified as slow progressors in our study had a low risk of RRT (about 25% within 5 years in CKD stage G5), while elderly with a fast progression still had much lower RRT risk than the younger patient of similar CKD stage.

Some previous studies have shown that both age and CKD stage influence the risk of RRT, but also that there is a strong interaction between them because of the competing risk of death [3, 17]. The two largest of these studies were performed in different clinical settings (O’Hare [17] used a cohort of American Veterans, whereas De Nicola [3, 18] used nephrology- referred patients in a geographical region in Italy). In the US-based study, the risk of mortality before RRT initiation was higher compared with the Italian study. The results of our study in Swedish nephrology-referred patients, however, demonstrated a high mortality before RRT initiation in line with the study by O’Hare [17]. It has been suggested that differences in RRT risk are attributed to the population background; general population cohorts have overall lower end-stage kidney disease risk as compared with specialized care cohorts [4, 19]. However, our results suggest that the risk of RRT and end-stage kidney disease does not only depend on general versus referred population but also on other factors. These factors could be underlying population mortality (there is a strong north-south cardiovascular disease risk gradient across Europe [20] which may explain the higher mortality before RRT in Sweden versus Italy), or they could instead reflect differences in attitude towards initiating dialysis in elderly individuals [21], timing of dialysis initiation [22] or referral pattern [23]. For example, a recent comparison of the performance of the kidney failure risk equation demonstrated that there were quite substantial differences in the risk for end-stage kidney disease between various cohorts in Europe and the US [24].

Decline in renal function has been associated with increased cardiovascular mortality [25] and initiation of RRT [26]. In younger individuals, low eGFR *per se* is more predictive of end-stage kidney disease since it is more often the result of a specific kidney disease as compared with elderly where it is more often a marker of age-related conditions [17]. Recently Coresh and coworkers demonstrated the importance of a rapid progression rate and suggested that a decline in GFR of 30% over two years could be used as an end-point in clinical trials [4]. Our study is in agreement with these data, and indicates that also in elderly individuals the progression rate influences the risk of RRT but not the competing risk of death to the same extent. However, the absolute risk of RRT is still extensively influenced by the competing risk of death in elderly individuals, also under the circumstance of a fast progression rate - in our data shown as a decreasing absolute risk difference with age.

It has been proposed that trajectories of renal function could be used to predict timely RRT planning [27]. In addition, early referral to a nephrologist is associated with better survival [24, 28] and more timely preparation for dialysis care. However, it is equally important that the resources of nephrology care are directed to where it is most needed. Our results indicate that continued follow-up at the nephrology unit is most important for younger CKD patients, especially those with a fast progression rate who also exhibits a high absolute risk of RRT start within 5 years. In elderly patients (>75 years of age) the 5-year risk of RRT is low-moderate also at advanced stages of CKD and the 5-year risk of RRT is especially low in those with a slow progression rate. Thus, elderly slow progressors in CKD stage 3b-4 who do not suffer any major metabolic disturbances could be referred to primary health care with the proviso that they should be returned to the nephrology clinic if they progress to CKD stage 5 where the risk of future RRT increases. In these elderly patients, timely access planning also has to be weighed against the risk of dying with a functioning fistula before or shortly after RRT initiation since mortality continues to be high also the year after dialysis start [29].

Our study has several strengths. It is the first study to use a national, representative cohort under stable nephrology care, where the treating nephrologists provided the primary renal diagnoses, and where clinical information was available both at inclusion and follow-up. Due to the use of linkages to national registries and the unique personal identity number of all Swedish citizens there were no losses of follow-up. The health-care system in Sweden is tax-funded, and there are no major differences in incidence of RRT throughout the country [30]. Our study also has some limitations. Although 96% of the nephrology units in Sweden send data to the SRR-CKD, it has been estimated that about 76% of all nephrology-referred CKD stage 4–5 patients are included in the registry [7]. In earlier CKD stages (3b), the completeness is unknown, since it is not mandatory to use the registry until eGFR drops below 30 ml/min/1.73 m^2^. However, registrations should be systematic within each clinic, and bias due to selection would be possible only if clinics registering patients already at CKD stage 3 differ in their RRT initiation policy or mortality from those who do not. Another limitation is that we excluded patients with less than two creatinine measurements or who died within one year. Thus, our results are only generalizable to patients under stable nephrology care for more than one year. When considering all patients referred to nephrology care, the risk of death before RRT initiation may be even higher. Furthermore, ACR was not measured in all the patients since it was not a mandatory variable in the registry until 2013. The associations between ACR, progression and mortality are well described [31]. However, in the sensitivity analysis in our study, adding ACR did not substantially affect our results regarding mortality. In addition, the progression rate would in itself consider some of the effect of ACR. Further research is needed to investigate whether adding progression rate as a predictor to the kidney failure risk equation could improve its performance and maybe explain some of the variation in incidence of RRT between the different CKD cohorts.

## Conclusions

In this nationwide registry study of 8,771 referred patients with CKD stage 3b-5, the risk of RRT was high in younger patients with fast initial progression rate. Furthermore, the cumulative incidence of RRT was generally low among elderly, slowly progressing patients even in advanced CKD stages. Thus, for planning, treating and preparing the right patients for RRT, following the slope of eGFR is important.

## Additional files

Additional file 1: STROBE_RRT incidence. Documentation of adherence to STROBE statement. (DOC 83 kb)
Additional file 2:
**Figure S1.** Description of study design. **Figure S2.** Five-year cause-specific probabilities of renal replacement therapy and death before renal replacement therapy for diabetic patients with a fast and slow progression rate stratified by age and chronic kidney disease stage. (PDF 100 kb)

## Abbreviations

ACR: Albumin Creatinine Ratio
BMI: Body Mass Index
CKD: Chronic Kidney Disease
CKD-EPI: Chronic Kidney Disease Epidemiology Collaboration
eGFR: Estimated Glomerular Filtration Rate
ESRD: End-Stage Renal Disease
RRT: Renal Replacement Therapy
SHR: Sub Hazard Ratio
SRR-CKD: Swedish Renal Registry - Chronic Kidney Disease
