# Supplementary material for: Low renal replacement therapy incidence among slowly progressing elderly chronic kidney disease patients referred to nephrology care: an observational study
Source: BMC Nephrol. 2017 Feb 10;18:59. doi: 10.1186/s12882-017-0473-1 (PMC5303237; doi:10.1186/s12882-017-0473-1)
Supplement: Additional file 1: — STROBE_RRT incidence. Documentation of adherence to STROBE statement. (DOC 83 kb) [file 12882_2017_473_MOESM1_ESM.doc]

STROBE Statement—checklist of items that should be included in reports of observational studies

|  | Item No | Recommendation |
| --- | --- | --- |
| **Title and abstract** | 1 | (*a*) Indicate the study’s design with a commonly used term in the title or the abstract - yes |
|  |  | (*b*) Provide in the abstract an informative and balanced summary of what was done and what was found -yes |
| Introduction | | |
| Background/rationale | 2 | Explain the scientific background and rationale for the investigation being reported - yes |
| Objectives | 3 | State specific objectives, including any prespecified hypotheses -yes |
| Methods | | |
| Study design | 4 | Present key elements of study design early in the paper |
| Setting | 5 | Describe the setting, locations, and relevant dates, including periods of recruitment, exposure, follow-up, and data collection – Information in Methods – study population and Flow chart (Figure 1) |
| Participants | 6 | (*a*) *Cohort study*—Give the eligibility criteria, and the sources and methods of selection of participants. Describe methods of follow-up. Yes - In Methods – study population |
|  |  | (*b*) *Cohort study*—For matched studies, give matching criteria and number of exposed and unexposed – Not matched |
| Variables | 7 | Clearly define all outcomes, exposures, predictors, potential confounders, and effect modifiers. Give diagnostic criteria, if applicable. Yes – variables and exposures defines in Renal function and Clinical measures section |
| Data sources/ measurement | 8* | For each variable of interest, give sources of data and details of methods of assessment (measurement). Describe comparability of assessment methods if there is more than one group. The measures are described in Methods section (clinical measures and renal function) |
| Bias | 9 | Describe any efforts to address potential sources of bias – Description in Discussion part (limitations) |
| Study size | 10 | Explain how the study size was arrived at. Yes (Figure 1, flow chart) |
| Quantitative variables | 11 | Explain how quantitative variables were handled in the analyses. If applicable, describe which groupings were chosen and why (Yes - Methods, statistics) |
| Statistical methods | 12 | (*a*) Describe all statistical methods, including those used to control for confounding (Yes, described in Methods statistics) |
|  |  | (*b*) Describe any methods used to examine subgroups and interactions (Explained in Statistics section) |
|  |  | (*c*) Explain how missing data were addressed (described in Statistics section) |
|  |  | (*d*) *Cohort study*—If applicable, explain how loss to follow-up was addressed (yes, registry follow up with no losses) |
|  |  | (*e*) Describe any sensitivity analyses (Described in statistics and results) |

Continued on next page

| Results | | |
| --- | --- | --- |
| Participants | 13* | (a) Report numbers of individuals at each stage of study—eg numbers potentially eligible, examined for eligibility, confirmed eligible, included in the study, completing follow-up, and analysed (Reported in Figure 1) |
|  |  | (b) Give reasons for non-participation at each stage (yes, see Figure 1) |
|  |  | (c) Consider use of a flow diagram (yes) |
| Descriptive data | 14* | (a) Give characteristics of study participants (eg demographic, clinical, social) and information on exposures and potential confounders (See Table 1) |
|  |  | (b) Indicate number of participants with missing data for each variable of interest (overview Table 1) |
|  |  | (c) *Cohort study*—Summarise follow-up time (eg, average and total amount) (Yes, in results section) |
| Outcome data | 15* | *Cohort study*—Report numbers of outcome events or summary measures over time (yes, in results) |
|  |  |  |
|  |  |  |
| Main results | 16 | (*a*) Give unadjusted estimates and, if applicable, confounder-adjusted estimates and their precision (eg, 95% confidence interval). Make clear which confounders were adjusted for and why they were included (Yes, in results and table 2) |
|  |  | (*b*) Report category boundaries when continuous variables were categorized |
|  |  | (*c*) If relevant, consider translating estimates of relative risk into absolute risk for a meaningful time period (yes, see Figure 4-5) |
| Other analyses | 17 | Report other analyses done—eg analyses of subgroups and interactions, and sensitivity analyses |
| Discussion | | |
| Key results | 18 | Summarise key results with reference to study objectives (Yes, in beginning of discussion) |
| Limitations | 19 | Discuss limitations of the study, taking into account sources of potential bias or imprecision. Discuss both direction and magnitude of any potential bias (yes) |
| Interpretation | 20 | Give a cautious overall interpretation of results considering objectives, limitations, multiplicity of analyses, results from similar studies, and other relevant evidence (yes) |
| Generalisability | 21 | Discuss the generalisability (external validity) of the study results (yes) |
| Other information | | |
| Funding | 22 | Give the source of funding and the role of the funders for the present study and, if applicable, for the original study on which the present article is based (reported) |

*Give information separately for cases and controls in case-control studies and, if applicable, for exposed and unexposed groups in cohort and cross-sectional studies.

**Note:** An Explanation and Elaboration article discusses each checklist item and gives methodological background and published examples of transparent reporting. The STROBE checklist is best used in conjunction with this article (freely available on the Web sites of PLoS Medicine at http://www.plosmedicine.org/, Annals of Internal Medicine at http://www.annals.org/, and Epidemiology at http://www.epidem.com/). Information on the STROBE Initiative is available at www.strobe-statement.org.
