# Supplementary material for: Low renal replacement therapy incidence among slowly progressing elderly chronic kidney disease patients referred to nephrology care: an observational study
Source: BMC Nephrol. 2017 Feb 10;18:59. doi: 10.1186/s12882-017-0473-1 (PMC5303237; doi:10.1186/s12882-017-0473-1)
Supplement: Additional file 2: — Figure S1. Description of study design. Figure S2. Five-year cause-specific probabilities of renal replacement therapy and death before renal replacement therapy for diabetic patients with a fast and slow progression rate stratified by age and chronic kidney disease stage. (PDF 100 kb) [file 12882_2017_473_MOESM2_ESM.pdf]

Figure S1. Description of study design.

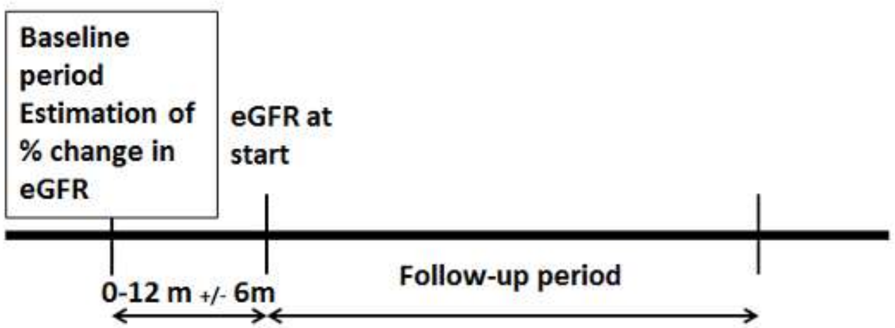

Figure S2. Five-year cause-specific probabilities of renal replacement therapy and death before renal replacement therapy for diabetic patients with a fast and slow progression rate stratified by age and chronic kidney disease stage

Cause-specific probability of RRT		Non - progressors			Progressors		
		Age			Age		
	CKD stage	<65	65-75	>75	<65	65-75	>75
	IIIb	15.9	10.5	7.5	32.2	22.0	16.0
	IV	29.8	20.3	14.7	54.7	39.8	30.0
	V	50.4	36.2	27.0	79.2	63.4	50.6

Cause-specific probability of death before RRT		Non - progressors			Progressors		
		Age			Age		
	CKD stage	<65	65-75	>75	<65	65-75	>75
	IIIb	15.0	33.1	58.8	18.7	40.1	67.7
	IV	14.1	31.3	56.4	17.5	38.0	65.2
	V	14.2	31.5	56.6	17.7	38.3	65.5
